# Quantitative dynamics of reversible platelet aggregation: mathematical modelling and experiments

**DOI:** 10.1038/s41598-019-42701-0

**Published:** 2019-04-17

**Authors:** Aleksandra A. Filkova, Alexey A. Martyanov, Andrei K. Garzon Dasgupta, Mikhail A. Panteleev, Anastasia N. Sveshnikova

**Affiliations:** 1 Faculty of Physics, Lomonosov Moscow State University, 1/2 Leninskie gory, Moscow, 119991 Russia; 2 Center for Theoretical Problems of Physicochemical Pharmacology, Russian Academy of Sciences, 4 Kosygina St, Moscow, 119991 Russia; 3 National Medical Research Centre of Pediatric Hematology, Oncology and Immunology named after Dmitry Rogachev, 1 Samory Mashela St, Moscow, 117198 Russia; 4 Faculty of Biological and Medical Physics, Moscow Institute of Physics and Technology, 9 Institutskii per., Dolgoprudnyi, 141700 Russia; 5 Department of Normal Physiology, Sechenov First Moscow State Medical University, 8/2 Trubetskaya St., Moscow, 119991 Russia

**Keywords:** Computational models, Platelets

## Abstract

Although reversible platelet aggregation observed in response to ADP stimulation in the presence of calcium is a well-known phenomenon, its mechanisms are not entirely clear. To study them, we developed a simple kinetic mass-action-law-based mathematical model to use it in combination with experiments. Light transmission platelet aggregometry (LTA) induced by ADP was performed for platelet-rich plasma or washed platelets using both conventional light transmission and aggregate size monitoring method based on optical density fluctuations. Parameter values of the model were determined by means of parameter estimation techniques implemented in COPASI software. The mathematical model was able to describe reversible platelet aggregation LTA curves without assuming changes in platelet aggregation parameters over time, but with the assumption that platelet can enter the aggregate only once. In the model, the mean size of platelet aggregates correlated with the solution transparency. This corresponded with flow cytometry analysis and with optical density fluctuations data on aggregate size. The predicted values of model parameters correlated with ADP concentration used in experiments. These data suggest that, at the start of the aggregation, when platelet integrins switch “on”, large unstable platelet aggregates are rapidly formed, which leads to an increase in light transmission. However, upon fragmentation of these aggregates, the probability of the post-aggregate platelets’ attachment to each other decreases preventing new aggregation and resulting in the reversible aggregation phenomenon.

## Introduction

Platelets are anuclear cells that circulate in blood for approximately 7–9 days after production in bone marrow. They are critically important elements of the hemostatic system that prevent blood loss upon vessel wall disruption. After a contact with damaged vessel wall, platelets become activated, adhere to the vessel and form an aggregate at the site of injury thus preventing blood leakage^1,2^. Both conditions of bleedings or thrombosis might be an outcome of platelet function disorders; thus, the assessment of platelet function is vital for diagnostics and therapy.

Since the time of its development in 1962 by Born and O’Brien^3–5^, light transmission aggregometry (LTA) has become the golden standard for investigation of platelet responses as well as the prime clinical diagnostic technique^1^. LTA is based on platelet suspension optical density (or turbidity) measurement. Platelet-rich plasma (PRP) or a suspension of washed platelets in buffer solution can be used, and magnetic stirrers are usually utilized to ensure platelet collisions with each other^6^.

**Figure 1:**
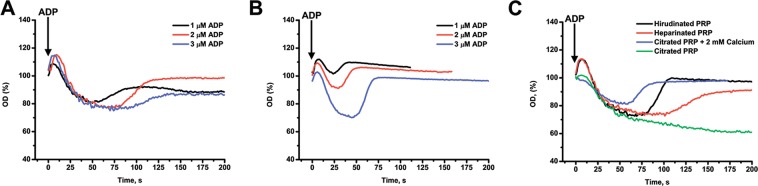
Aggregation of platelets in response to ADP. (**А**) Activation of heparinized platelet rich plasma by various concentrations of ADP; (**B**) Activation of washed platelets by the same concentrations of ADP as on panel (**A**) in the presence of fibrinogen (200 µg/ml). The aggregation response for washed platelets is always weaker than for platelets in PRP; (**С**) Activation of heparinized or hirudinated (with calcium ions in the solution), citrated (without calcium) or citrated upon recalcification (with calcium) platelet rich plasma induced by ADP (1.25 µM). The aggregation in citrated PRP without calcium is irreversible. Typical results out of n = 10 different donors.

After addition of an agonist to the suspension, platelets get activated and change shape^7–9^. This is commonly believed to be the reason for the initial increase in optical density after stimulation (Fig. 1). The formation of platelet aggregates occurs due to agonist-stimulated activation of the major platelet integrin α_IIb_β_3_. In its active form, integrin α_IIb_β_3_ binds molecules of fibrinogen from the solution, and in this way provides “bridges” between platelets. Via α_IIb_β_3_-fibrinogen-α_IIb_β_3_ bridges platelets form aggregates^1–3^. Aggregation of platelets makes cell suspension less turbid (decreases optical density) because the number of light-scattering particles in the suspension decreases^4,6^.

Although aggregometry is a routine straightforward technique, direct interpretation of its results is lacking. One of the unexplained phenomena is that platelet aggregation in response to the main activator ADP seems to be weaker in the presence of calcium ions in the solution (which is the correct physiological condition) than without them^10–14^ (Fig. 1). Platelet aggregation in these conditions is always reversible (Fig. 1A,B). One of the possible explanations is the inactivation of α_IIb_β_3_ that leads to platelet aggregate disassembly^6,9,13,15^. On the other hand, the breakdown of platelet aggregates could originate from some mechanistic reasons, such as turbulent flows in the aggregometric cuvette^1,2,16,17^. Understanding of these processes could improve the diagnostic potential of LTA.

To obtain platelet functional parameters from the complex shapes of the aggregation curves, a computational modelling approach could be utilized. The computational approach most widely used for particle aggregation tasks is Smoluchowski coagulation-fragmentation equation^18^, where some fragmentation and coagulation “kernels” are constructed and then solved by Smoluchowski equation^18–23^. Although the Smoluchowski approach could be the most “correct” one, it is quite computationally expensive when solved with deterministic methods, and still contain too many unknown parameters when solved with stochastic methods^24,25^ and, therefore, computational modelling of aggregometry data with Smoluchowski equation could not be readily performed alongside with personalized parameter estimation yet^26^. Another commonly used approach is the lattice models^27,28^, which is based on a construction of a lattice with a particle in each cell of the mesh. The aggregation is modelled by the energy-profit-driven movement of particles between the cells^27^. Although lattice-based models are useful, their complexity and computational cost are too high for three-dimensional mesh, which should be applied for aggregometry modelling. The most abundant are simplistic models, based on mass-action laws of kinetics with included description of particles clustering and aggregation^29,30^. However, most of them were developed for irreversible aggregation, and therefore focus on the level of aggregation as the main parameter, and pre-define which parameter values should depend on the level of activation^31,32^.

Here we suggest that the crucial limitation of the existing models is the external introduction of the effect of activator on the laws of aggregation, and we propose to deduce parameters of platelet activation from the experimental data by means of parameter estimation techniques. In the current study, this approach allowed us to describe experimental data on the reversible platelet aggregation and conclude that the origin of the reversibility lies in the instability of large aggregates rather than in the loss of aggregatory ability as a function of time. This conclusion was confirmed by flow cytometry and optical density fluctuations analysis^33^ of the aggregate size distribution in the course of platelet aggregation.

## Results

### Construction of the mathematical model of platelet aggregation in response to ADP

Platelet aggregation in response to ADP in the presence of calcium ions in the medium is reversible (Fig. 1)^34,35^. There was a dose-dependent increase of reversible aggregation in heparinized or hirudinated platelet rich plasma (PRP) in response to ADP, with transition reversible/irreversible aggregation at 1.25–5 µM (Fig. 1A). Washed platelets with 200 µg/ml fibrinogen reacted similarly (Fig. 1B). Calcium ions chelation by sodium citrate led to an irreversible aggregation (Fig. 1C).

**Figure 2:**
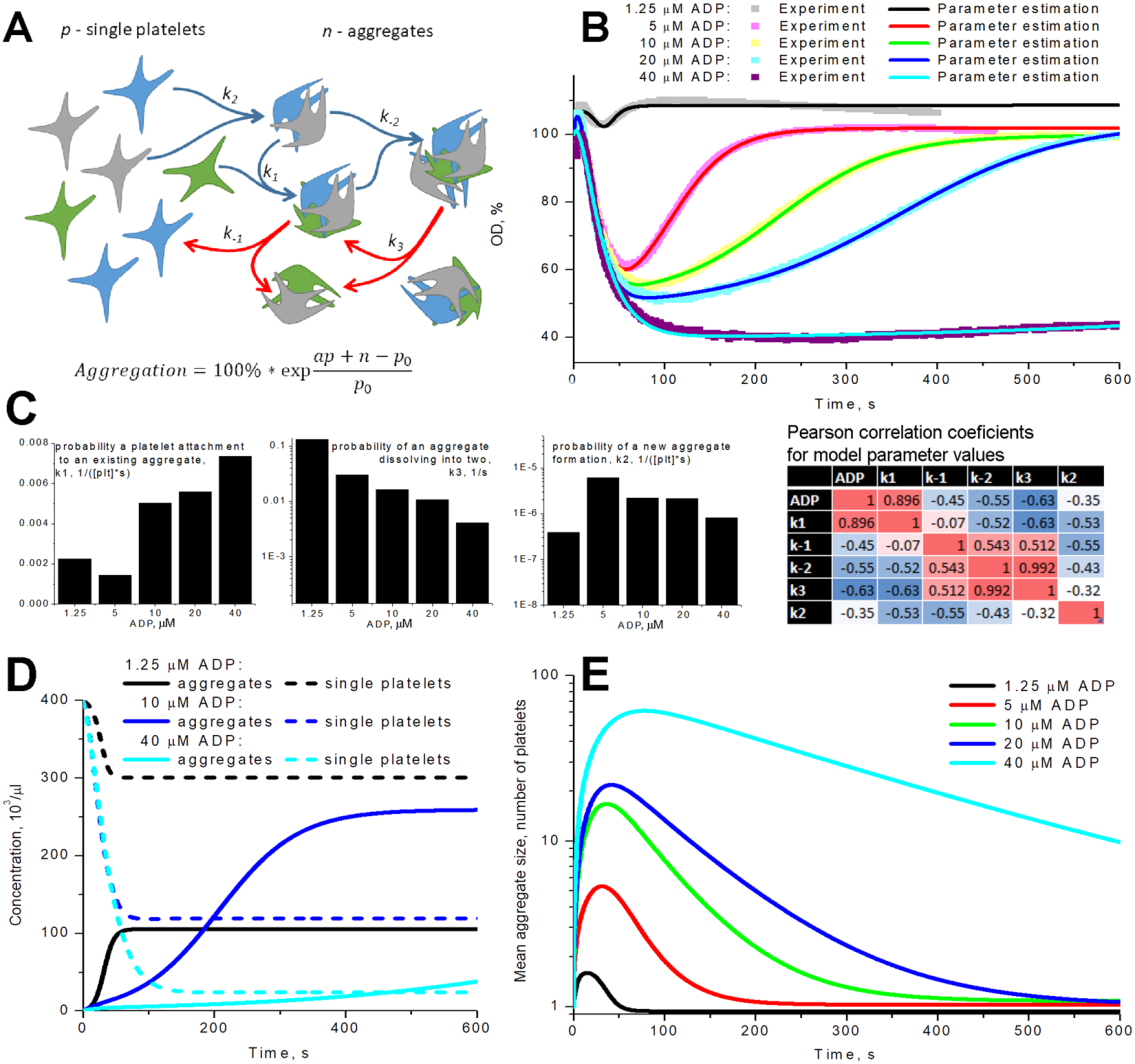
Mathematical model for platelet aggregometry. (**A**) Cartoon of the mathematical model of platelet aggregation, which is based on mass action kinetics (see text) for reactions between aggregates (n) and single platelets (p); (**B–E**) the estimation of five model parameters was conducted automatically by means of five different parameter estimation techniques implemented in COPASI software (see text). For each set of experimental data, parameters of the models were estimated independently; (**B**) washed platelets, stimulation with 1.25, 5, 10, 20 or 40 µM of ADP, experimental curves were taken from the same blood sample of the same donor, the presented single curves are typical out of n = 3 sets for this donor and this donor expose typical results out of n = 10 different donors; the complete sets of parameter values for ADP (1.25-5-10-20-40) were: k_1_ = (2.2, 1.4, 5, 5.6, 7.3)*10^−3^ 1/([plt]*s), k_−1_ = (0.67, 0.13, 0.59, 0.54, 0.18) 1/s, k_−2_ = (1.2, 0.1, 0.063, 0.036, 0.023)*10^−3^ 1/([plt]*s), k_3_ = (13, 3, 1.6, 1, 0.4)*10^−2^ 1/s, k_2_ = (4, 60, 22, 21, 8.1)*10^−7^ 1/([plt]*s), a = (1.08, 1.16, 1.17, 1.19, 1.14), p_0_ = (400, 405, 400, 400, 400) [plt]; (**C**) dependence of parameter values on ADP concentration used in corresponding experiments and a table of Pearson correlation coefficients for this set of data; (**D**) calculated time-course of the concentration of single platelets (p) and aggregates (n) for the same models as on panel (B,E) calculated time-course of the mean size of aggregate (in platelets) for the same models as on panel (B).

Movement of individual platelets and aggregates in the cuvette of the aggregometer could not be described analytically and requires excessive computer time to model numerically^17^. However, the rate of hydrodynamic flows in an aggregometry cuvette without platelets could be calculated (Fig. S1). Our calculations together with previous reports^36^ show that there is no significant dependence of the shear rate on the rpm in the working range of the aggregometer (800–1200 rpm). We also confirmed these results experimentally (Fig. S2). Surprisingly, it appears that neither stirring rate, nor the measurement height significantly affect the behaviour of the aggregation curve (Fig. S2). Accordingly, we assumed that the sole purpose of fluid flows in the cuvette is an enhancement of cell-cell interactions with each other and with aggregates. The event of aggregate formation then can be described by a probability constant. In response to ADP fibrinogen binding to a single platelet increases step-like (Fig. S3), so we can assume that the probabilities of platelet attachment to each other do not change with time. In case of secondary platelet activation, for example, due to thromboxane A2 synthesis^37^, the assumption of constant attachment probabilities will not hold and in such case the model will not be applicable. In the real solution, the platelets can be found in numerous states: single platelets with or without filopodia and aggregates of various size and density. However, the simplest approximation is that a significant difference exists between the states of single and aggregated platelets^16^. Then the simplest mathematical model of platelet aggregation in suspension will be:

1 $$\frac{dp}{dt}=-\,{k}_{1}np-2{k}_{2}{p}^{2}+{k}_{-1}n,$$

2 $$\frac{dn}{dt}={k}_{2}{p}^{2}-{k}_{-2}{n}^{2}+{k}_{3}n,$$

where *p* is the concentration of single platelets, *n* is the concentration of aggregates, *k*_2_ is the probability of new aggregate formation from two single platelets, *k*_1_ is the probability of another platelet attachment to an existing aggregate, *k*_*−1*_ is the probability of single platelet detachment from an aggregate, *k*_*−2*_ is the probability of formation of one aggregate from two existing ones and *k*_3_ is the probability of an aggregate fragmenting into two (Fig. 2A). All probabilities *k*_i_ theoretically depend on the average size of aggregates, which can be calculated as

3 $$s=\frac{{p}_{0}-p}{n},$$

where *p*_0_ is the initial concentration of platelets. The eq. (3) could be considered as a mass conservation law. Qualitative analysis of the model (see supplement) revealed that a quasi-oscillatory regime could be found for it and, thus, the reversible aggregometry data can be described by this model without variation of the model parameters with time. From the experimental point of view, this means that the observed reverse course of platelet aggregation (Fig. 1) does not necessarily mean deactivation of platelets.

### Estimation of model parameters from LTA data

We then proceeded to the estimation of the model parameters from the experimental data. We used five different parameter estimation techniques for each experimental dataset to ensure unbiased parameter estimation. In Fig. 2B, typical parameter estimation results are shown. The output parameter of the aggregometer (percentage of aggregation) could be compared with the concentration of light-scattering particles in the solution according to the following equation^30^:

4 $$Aggregation=100 \% \,\ast \,\exp \frac{ap+n-{p}_{0}}{{p}_{0}},$$

where parameter *a* is introduced to describe platelet shape change upon activation, it is considered to be linearly increasing with time in the first two seconds of the simulation.

Analysis of the experimental data shows that ADP concentration influences all parameters of the model (Fig. 2C with legend), with the probability of aggregate formation (k_1_) increasing with ADP concentration increase for the particular donor, and the parameters of single aggregate stability (k_−2_, k_3_) decreasing with it (note that weak response to 1.25 µM ADP slightly falls out of these trends). With the mathematical model presented here, we have tried to perform comparison between platelet aggregation in PRP and in buffer solution (Fig. S4). However, we could not reach any reliable conclusion except that the parameter of aggregate stability (k_3_) increases for platelet-rich plasma. Analysis of the validated model revealed that the aggregate size increased from 1 to 2–10 in the first minute of experiment and then dropped to 2–1 (Fig. 2D,E). Meanwhile the corresponding concentration of aggregates increases constantly with time and the corresponding concentration of free platelets decreases with it (Fig. 2D,E).

### Aggregate size distribution over the course of aggregation

**Figure 3:**
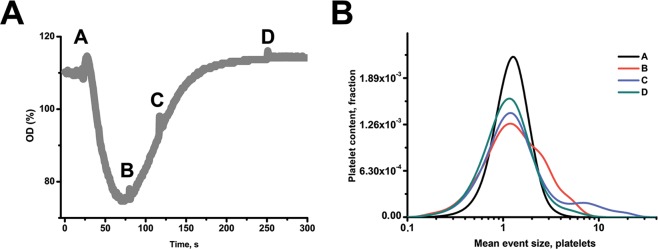
Flow cytometry estimation of aggregate sizes during aggregometry test. Aggregation of washed loaded with Fura Red human platelets in Tyrode’s calcium buffer (3·10^5^/µl) in the presence of 200 µg/ml human fibrinogen was stimulated with 2.5 µM ADP. Samples of the aggregating mixture were taken at points indicated as (**A–D**) on the aggregation curve (A) and immediately analyzed by flow cytometry. Relative platelet size was measured as ratio of signal in FL-3 (Fura-Red) and mean FL-3 fluorescence of single platelets. One typical aggregation curves out of n = 3 for this donor, similar results were obtained for n = 2 another donors.

The conclusion of the model that aggregation peak corresponds to the peak in aggregate size, while assuming that the adhering properties of single platelets do not change, can be tested by means of flow cytometry. The prediction that the aggregation peak corresponds to an increase in aggregate size, was investigated by comparison between side scattering-forward scattering dot plots of samples taken from the aggregometer (Fig. 3, S5). The distribution of fluorescence intensity (that we assume to mimic the distribution of aggregate sizes) appeared to have several characteristic ranges. At the aggregation curve maximum, about 60% of platelets remained single, 25% were in small aggregates of 2–4 cells, and 15% were in large aggregates of 4–8 cells. After that, the small aggregates began to disappear, while the large ones seemed even to increase in size (up to 10–20 cells). The large aggregates disappeared completely over the further course of disaggregation (Fig. 3), and only a small number of smaller aggregates remained. To support this conclusion, we also performed dark-field microscopy of samples from the aggregometry cuvette at various time points and confirmed that large aggregates appear in the cuvette only at the peak of aggregation (Fig. S6). In addition, we have extended the model to describe dynamics of aggregates of various sizes. It contains the same number of unknown parameters, but increased number of variables. This model demonstrates, that at the aggregational peak most platelets are in aggregates of size 2–10 and only a few in aggregates conformed of >10 cells. All these data lead us to the conclusion that size distribution of platelets is only significant in small range (1–10) and so does not dramatically affect the model, described in (1–3) (Fig. S7).

### Simultaneous measurement of platelet aggregation and mean aggregate size

**Figure 4:**
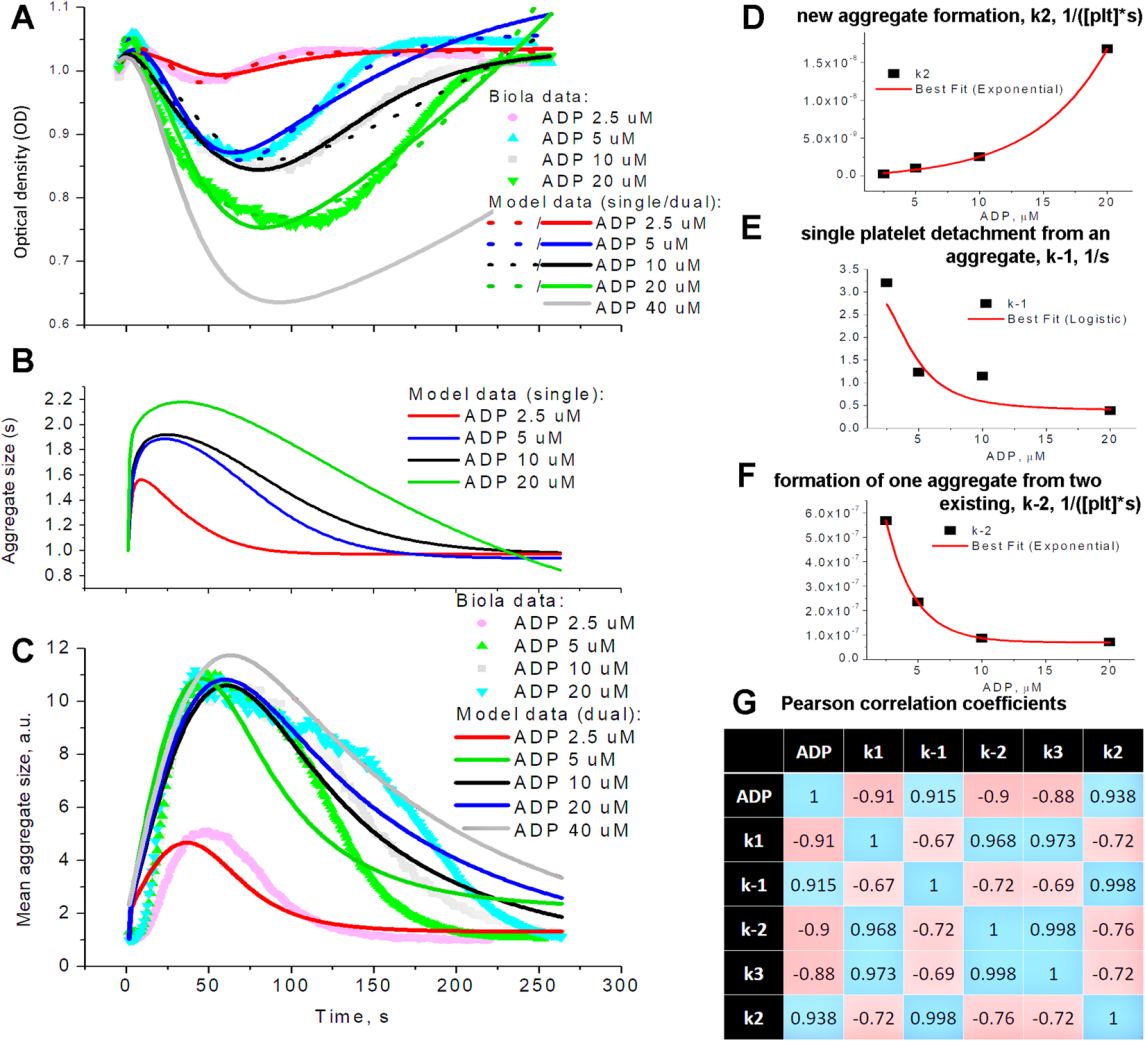
Parameter estimation for platelet aggregometry data obtained by Biola. Estimation of five model parameters and initial platelet concentration (Table 1) was conducted automatically either as in Fig. 2 (“single”), or with both light transmission curve and mean aggregate size as input experimental data (“dual”). For each set of experimental data, parameters of the models were estimated independently. (**A**) washed platelets, stimulation with 2.5, 5, 10 or 20 µM of ADP, experimental curves were taken from the same blood sample of the same donor, typical experiment out of n = 3 for this donor and out of n = 4 different donors; (**B**) calculated time-course of the mean size of aggregate (in platelets) for the same models (“single”) as on panel (A); (**C**) experimental and calculated time-course of the mean size of aggregate for the same conditions (models “dual”) as on panel (A); (**D–F**) Correlation of the probability of (**D**) new aggregate formation, (**E**) single platelet detachment from an aggregate, (**F**) formation of one cluster from two existing, for dataset from panel (A), models “dual”. (**G**) Table of Pearson correlation coefficients for this set of data (models “single”).

Another support for the predicted aggregate size dynamics comes from a version of the LTA technique, where the fluctuations of the light transmission signal are used to estimate a relative size of light-dissipating particles^38^. The LTA signal received in Biola aggregometer possesses the same features as all other LTA data (Fig. 4A for standard platelet concentration, Fig. S8 for low platelet concentration (100 000/μl) and high activator concentrations (20–50 μM)) and could be successfully described by the model (1)-(3) (Fig. 4A, dashed curves). Predicted by the corresponding models time-courses for the aggregate size are given in Fig. 4B. Calculated by the Biola device time-course of mean size is given in Fig. 4C. It could be seen by comparison of panels B and C that although the main features of the experimental and theoretical size distribution are similar, the particulars are different. The origin of such discrepancy could be the unknown value for the platelet concentration in the solution. As the next step, we allowed it to be estimated by the model, where both aggregation data and the mean size data were described simultaneously (“dual” model in Fig. 4). It appeared that the model could successfully describe both types of data, although with less accuracy (Fig. 4, Table 1). However, only the parameters of the “dual” model highly correlate with ADP concentration (Fig. 4D,F) and with each other (Fig. 4G). This suggests that they are not independent, but rather represent a combination of a smaller number of some basic functions of platelet activation.

**Table 1:** Automatically assessed model parameters for experimental datasets given in Fig. 4.

Parameter	2.5 µM ADP	5 µM ADP	10 µM ADP	20 µM ADP	2.5 µM ADP	5 µM ADP	10 µM ADP	20 µM ADP	40 µM ADP
	single	single	single	single	dual	dual	dual	dual	dual
probability of a platelet attachment to an existing aggregate, k_1_, 1/([plt]*s)	8.6*10^−4^	6.8*10^−4^	5.2*10^−4^	4.3*10^−4^	4.8*10^−6^	4.6*10^−6^	1.3*10^−5^	1.8*10^−5^	1*10^−5^
probability of single platelet detachment from an aggregate, k_−1_, 1/s	1.0*10^−9^	4.0*10^−16^	3.5*10^−15^	1.0*10^−4^	3.2	1.22	1.15	0.39	0.2
probability of one aggregate formation from two existing, k_−2_, 1/([plt]*s)	6.7*10^−4^	3.8*10^−4^	2.3*10^−4^	7.6*10^−7^	5.7*10^−7^	2.5*10^−7^	8.7*10^−8^	7.1*10^−8^	6.5*10^−8^
probability of an aggregate fragmenting into two, k_3_, 1/s	0.07	0.03	0.02	0.005	0.03	0.017	0.009	0.007	0.0035
probability of a new aggregate formation, k_2_, 1/([plt]*s)	3.8*10^−5^	3.9*10^−5^	5.1*10^−5^	2.7*10^−4^	2.5*10^−10^	1.0*10^−9^	2.5*10^−9^	1.7*10^−8^	3*10^−8^
initial concentration of platelets, p_0_, [plt]	100	100	100	100	742779	433994	125561	54038	35000

To assess comparative aggregation potential of single platelets during platelet aggregation, we performed aggregation test of Fura-Red loaded washed human platelets in presence of FITC-labelled fibrinogen (Figs S9 and S10). The relative aggregation potential was assessed as the ratio of fluorescence of fibrinogen-FITC and Fura-Red for each event. The fibrinogen binding to single platelets increases upon activation and was stable over time, while fibrinogen binding to aggregated platelets is significantly higher. Together these data indicate that the process of platelet disaggregation is determined by some instability in bonds between platelets in an aggregate.

## Discussion

In the present study, we developed a mathematical model of reversible platelet aggregation which incorporated a novel mechanism of disaggregation, could be used for clinical parameter estimation, and revealed a dependence of the aggregation and aggregate stability parameters on ADP concentration. The experiments performed to test these predictions added several more pieces of information, including the dynamics of aggregate size distribution. The study altogether suggested that the loss of the ability to form aggregates is maybe not the cause, but rather the consequence of disaggregation, that is supported by experiments with fibrinogen binding to single platelets (Fig. S3).

The mathematical model (eqs 1 and 2, Fig. 2A) described processes of aggregate formation and fragmenting without taking into consideration possible heterogeneity of aggregates in size and other aspects. The parameters of the model were derived for each donor from the given experimental data by means of parameter estimation techniques. Assessment of additional experimental data is to be done. Changing between PRP and washed platelets also has an impact on the parameter values. There is even a difference in platelet aggregation in PRP with hirudin or heparin (Fig. 1). There are several possibilities for the origin of the more pronounced aggregation in platelet rich plasma compared to the washed platelet suspension. To test the most obvious ones we have adjusted platelet concentrations and fibrinogen concentration in the washed platelet suspension to that of PRP levels (Fig. S11). However, platelet aggregation still did not reach the PRP levels. Other possibilities like addition of platelet poor plasma to washed platelets could be tested in the future. Never the less, we expect that, given enough data, it would be possible to establish an explicit connection between values of the model parameters and features of the sample (ADP concentration, plasma presence, device features).

ADP induced aggregation in the presence of free calcium ions is clearly distinct from the one in the presence of calcium ion chelator (citrate). Furthermore, PRP recalcification resulted in a reversible aggregation, which was observed in hirudinated of heparinated PRP (Fig. 1C). However, some differences in aggregation between hirudinated and heparinated PRP could not be explained so readily. It has been reported that heparin is capable of inducing the micro-aggregate formation in PRP, which are potentiating further platelet aggregation as well as false-positive LTA results^39^. Furthermore, it has been demonstrated that heparin is a positive mediator of platelet integrin function^40^ and thus, in heparinized PRP platelet aggregation might be additionally potentiated. Based on this, it can be claimed that hirudin is a more attractive option for aggregation evaluation in PRP.

The first mathematical model prediction is that the features of the aggregating particles (platelets or aggregates) do not change with time by themselves. The reverse course of aggregation originates from the transition of platelets between single and aggregated states. These prediction is partly supported by flow cytometry experiments performed here where the level of fibrinogen binding to single platelets was shown to be unchanged during aggregation. The increase in fibrinogen binding was observed for the platelet population as a whole, and was associated with disappearance of large aggregates (Fig. 3). Similar experiments with diluted platelets also demonstrate that platelet integrins do not inactivate within minutes (Fig. S3).

The mathematical model presented here is exceptionally simple and thus is not capable to describe fine features of processes in the aggregometer. As could be deduced from data in Fig. 3, a broad range of sizes of aggregates are present in the mixture during aggregation, while only the mean aggregate size is calculated in the model. The non-uniformity of aggregate size distribution has been shown several decades ago experimentally as well as theoretically^16,17^, but the results of the present study suggest that this distribution is not essential for correct description of platelet aggregation. An extension of the model proposed here to assess the dynamics of aggregates of different sizes supported this conclusion (Fig. S7). Another limitation, avoided in the majority of more profound mathematical models, is the inequality of the activation of platelets situated in different parts of the cuvette due to varying shear stresses^16,17^. In future work, we propose to derive intrinsic parameters of activation from experimental data by means of parameter estimation techniques instead of introducing them externally into the model, and the shear stress will be linked to one of the parameters. Another limitation of the model is that on long scale (more than ten minutes) the features of platelet activation start to change and the model with fixed parameter values cannot describe the data any more. To avoid this limitation either long-scale experiments should not be considered, or additional parameters should be introduced into the model.

## Materials and Methods

### Reagents

The sources of the following materials were as follows: ADP, PGI_2_, HEPES, bovine serum albumin, apyrase grade VII, fibrinogen (Sigma-Aldrich, St Louis, MO). Li-heparin and Na-citrate containing blood collection tubes (Vacuette®, Greiner Bio-One GmbH, Austria). Acid-citrate-dextrose (ACD) anticoagulant solution was prepared by dissolving trisodium citrate dehydrate (85 mM), citric acid monohydrate (66.6 mM), and anhydrous D(+)glucose (111 mM) in deionized water, pH was adjusted to 4.5. One volume of ACD is required for six volumes of blood. Tyrode’s albumin buffer contains NaCl, KCl, NaHCO3, NaH2PO4, MgCl2•6H2O, CaCl2•6H2O, HEPES (N-[2-hydroxyethyl]piperazine-N′-[2-ethanesulfonic acid]), bovine serum albumin (BSA), anhydrous D(+)glucose in deionized water. The pH was adjusted to 7.35. All salts for buffer preparation were from (Sigma-Aldrich, St Louis, MO).

### Blood collection and platelet isolation

Healthy volunteers, both men and women aged between 18 and 39 years were recruited into the study. Investigations were performed in accordance with the Declaration of Helsinki and approved by CTP PCP RAS ethic committee, written informed consent was obtained from all donors. For platelet rich plasma, blood was collected into 9 ml tubes containing 3,8% sodium citrate (1:9 vol/vol) or into 9 ml tubes containing Li-heparin at 1600 g for 15 min as described previously^41^. Platelet washing procedure was described previously^42^. Briefly, blood was collected into 15 ml tubes containing 2.1 ml ACD. Platelets were purified by double centrifugation in the presence of PGI_2_, or PGI_2_ and heparin in Tyrode’s albumin buffer as described previously^42^. Final solution of washed platelets in Tyrode’s buffer was supplemented with apyrase (0.1 U/mL) and rested for at least 30 minutes before the start of the experiments. All steps of washing procedure and all aggregometry experiments were conducted at 37 °C. PRP was diluted by PPP in order to achieve equal concentrations in all experiments. 27 healthy volunteers age from 18 to 39 (median age 25, 16 men, 11 women) were enrolled.

### Aggregometry

Platelet aggregation was performed using Chrono-Log 490 and Biola LA-230 turbi-diametric aggregometers. Experiments were conducted in 250 μL (Chrono-Log) or 300 μL (Biola) aliquots of PRP or washed platelets. The platelet suspension was mixed by a stirrer with 800 rpm. ADP was added at various concentrations as the platelet activator. Washed platelets were pre-incubated with 200 µg/ml fibrinogen or without it for control sample. Before ADP activation of PRP, fibrinogen was not added. Tyrode’s albumin buffer was used as a reference for washed platelets, and platelet poor plasma was used as a reference for PRP. Before measurements platelets were incubated at 37 °C. An optical signal was recorded every 0.5 s for Chrono-Log and 1 s for Biola.

### Aggregate size analysis by Biola

Aggregate size analysis was performed using Biola LA-230 turbi-diametric aggregometer. The method is based on the Gabbasov’method of light transmission fluctuations^33,38^. The fluctuations arise due to a change in the number of particles in the suspension. The basic parameter of the method is calculated by the formula:

$${(\frac{\sigma }{I})}^{2}=\theta \frac{{\sum }_{n}{K}_{n}^{2}{N}_{n}n{R}_{n}}{{\sum }_{n}{N}_{n}n},$$

where $\sigma$ is the average square deviation of light transmission, *I*–the mean value of light transmission, $\theta$ – the overall volume of platelets, *K*- the efficiency of scattering, *N* – the number of platelets, *R*– the radius of aggregate of $n$ - platelets.

### Extended model of platelet aggregation

Extended moodel of platelet aggregation is based on mass-action laws o kinetics. It consists of N differential equations each of which represents dynamics of aggregate of size $j$.

$$\begin{array}{rcl}\frac{dp}{dt} & = & -{k}_{1}p\sum _{i=2}^{N-1}[i]-2{k}_{2}{p}^{2}+{k}_{-1}(\sum _{i=2}^{N}[i]+[2])\\ \cdots  &  & \\ \frac{d[j]}{dt} & = & {k}_{-2}(\sum _{i=2}^{j/2}[i][j-i]-[j]\sum _{i=2}^{N-j}[i])\,+\,{k}_{3}(\sum _{i=j+2}^{N}[i]-(\lfloor \frac{j}{2}\rfloor -1)[j])\\  &  & -{k}_{1}p([j]-[j-1])+{k}_{-1}([j+1]-[j])\end{array}$$

Number of equations = N

where [*j*] is concentration of an aggregate of size *j* and other parameters are the same as described in Section 2.1.

### Parameter estimation and model solution

Construction of computational model was based on the principles described in the Results section. The set of ordinary differential equations was integrated using the LSODA method implemented in COPASI software^43^. The method LSODA^44^ is the numerical method to solve a system of ordinary differential equations with an ability to switch between explicit and implicit methods, which allows rapid integration of stiff problems (the equation dx/dt = ax presents a stiff problem). The COPASI (COmplex PAthway Simulator, copasi.org) is an open-source free software, where several methods for numerical integration and investigation of a system of ordinary differential equations are implemented. Model parameters were assesed from experimental data by means of the following teqniques implemented in COPASI software: Evolutionary Strategy (SRES)^45^, Particle Swarm^46^, Random Search, Hooke&Jeeves^47^ or Levenberg-Marquardt^48^.

## Supplementary information

Dataset 1
