# Supplementary material for: Quantitative dynamics of reversible platelet aggregation: mathematical modelling and experiments
Source: Sci Rep. 2019 Apr 17;9:6217. doi: 10.1038/s41598-019-42701-0 (PMC6470167; doi:10.1038/s41598-019-42701-0)
Supplement: Supplementary file 1 — Dataset 1 [file 41598_2019_42701_MOESM1_ESM.pdf]

Supporting information.

Title: Quantitative dynamics of reversible platelet aggregation: mathematical modelling and experiments

Authors: Aleksandra A. Filkova[1-2], Alexey A. Martyanov[1-3], Andrei D. K. Garzon[1], Mikhail A. Panteleev[1-4] and Anastasia N. Sveshnikova[1-3,5],

Corresponding author: Anastasia N. Sveshnikova, e-mail: a.sveshnikova@physics.mus.ru

1. Details of the model investigation.

At the beginning as the simplest assumption we state that k_i–s do not depend on *s*. Then the steady state of the system can be formulated analytically:

$$\begin{cases} p = \dfrac{k_1 k_{-1} - k_1 k_3 + \sqrt{-4k_2 k_{-1}^2 k_{-2} + k_1^2 k_3^2 + 8k_3 k_{-2} k_2 k_{-1}}}{k_1^2 + 4k_2 k_{-2}} \\ n = \dfrac{2k_2 p^2}{k_{-1} - k_1 p} \end{cases} \quad (4)$$

It should be noted that while the corresponding to steady state system of equations has four solutions, only one of them is positive and thus has biological meaning.

The stability of the system around steady state is determined by the eigenvalues of the corresponding Jacobian:

$$J = \begin{pmatrix} -k_1 n - 4k_2 p & 2k_2 p \\ -k_1 p + k_{-1} & -2k_{-2} n + k_3 \end{pmatrix} = \begin{pmatrix} j_{11} & j_{12} \\ j_{21} & j_{22} \end{pmatrix} \quad (5)$$

Here j_{11} is always negative, while j_{22} could be positive in cases of high k_3 values, j_{12} and j_{21} are always positive. This means that the system can have a steady state with the stability type “stable focus”, which could describe the reversible aggregometry data.

2. Role of hydrodynamic flows in platelet aggregation

In order to investigate the flow behaviour in the aggregometry cuvette we theoretically analyzed flows in an aggregometry cuvette by means of a computational model constructed in FlowVision CFD Software (https://flowvision.ru/). The model is based on Navier-Stokes equation, continuity equation and equations of standard k-epsilon turbulence model for flow of viscous fluid with the following assumed parameters for platelet-rich plasma. Physical parameters of liquid were obtained from literature (1) . To imitate the stirrer, the boundary conditions at the bottom of the cuvette were set as a tangential spin with frequency equal to frequency of the stirrer. According to the performed calculations (Fig. S1), the flows in the cuvette are mostly laminar, the flow velocities equal 0-10 sm/s and do not significantly change and, consequently, the shear rate also does not significantly change throughout the cuvette. The velocity and shear rate of the flow exhibit a little dependence on the rate of the stirrer rotation in the working range of aggregometer (800-1200 rpm) (Fig. S1B).

The role of the hydrodynamic flow in the aggregometry results could also be investigated experimentally. We have conducted experiments with rpm 800-1200 (Fig. S2A). Taking into account that the flow in the cuvette depends on the distance from the stirrer, we also analysed aggregation on different levels of the same aggregometry cuvette (Fig. S2B). In addition, keeping in mind that the adhesional ability of von Willebrand factor is strongly dependent on shear rate (2), we performed experiments with addition of a high concentration of vWF (Fig. S2C). We have performed parameter estimation for all described above experimental data. The parameter estimation show that the slight difference in experimental data

cause a difference within an order of magnitude in parameter values without correlation with rpm. With changes in height, the concentration of platelets decrease, together with the propabilities of aggregate formation (k1 and k-2).

Parameters for Fig. S2	800 rpm	900 rpm	1000 rpm	1200 rpm	1 μM ADP St. height	1 μM ADP +0.3cm	2 μM ADP St. height	2 μM ADP +0.3cm
probability of a platelet attachment to an existing aggregate, k_1, 1/([plt]*s)	$1*10^{-5}$	$5.6*10^{-6}$	$2.2*10^{-4}$	$2*10^{-4}$	$5.1*10^{-5}$	$1*10^{-6}$	$6.7*10^{-5}$	$4.6*10^{-6}$
probability of single platelet detachment from an aggregate, k_{-1}, 1/s	$9.2*10^{-13}$	$7.3*10^{-15}$	$6.7*10^{-7}$	$4.2*10^{-7}$	$4.1*10^{-11}$	$2.6*10^{-14}$	$3.2*10^{-16}$	$1.2*10^{-15}$
probability of one aggregate formation from two existing, k_{-2}, 1/([plt]*s)	$3*10^{-5}$	$2.5*10^{-5}$	$8.6*10^{-5}$	$7.4*10^{-5}$	$3.1*10^{-5}$	$1.1*10^{-5}$	$3.3*10^{-5}$	$9.0*10^{-6}$
probability of an aggregate fragmenting into two, k_3, 1/s	$2.9*10^{-2}$	$2.8*10^{-2}$	$12*10^{-2}$	$12*10^{-2}$	$4.7*10^{-2}$	$0.9*10^{-2}$	$3.5*10^{-2}$	$8.6*10^{-3}$
probability of a new aggregate formation, k_2, 1/([plt]*s)	$2.2*10^{-5}$	$2.3*10^{-5}$	$7.7*10^{-7}$	$1.3*10^{-6}$	$1.1*10^{-6}$	$1.4*10^{-5}$	$1.1*10^{-6}$	$1.2*10^{-5}$
impact of shape change, a, dimensionless	0.6	0.5	0.3	0.23	0.2	0.7	0.1	0.6
initial concentration of platelets, $p_{0,}$ [plt]	965	1209	1405	1450	1450	991	1105	917

Together these results demonstrate that the shear rates in the aggregometry cuvette are low (< 100 s^{-1}), these rates do not change with rpm and cannot activate platelets or plasma proteins. However, the measurement height of the aggregometry cuvette is important for the reproducibility of results.

3. An extended model of platelet aggregation

The mathematical model of platelet aggregation (1-3) contains only two variables - single platelets and aggregates, and therefore it cannot predict the distribution of aggregates by size. We constructed a detailed mathematical model which considers aggregates that are formed with different number of platelets.

$$\frac{dp}{dt} = -k_1 p \sum_{i=2}^{N-1} [i] - 2k_2 p^2 + k_{-1} (\sum_{i=2}^{N} [i] + [2])$$

$$\frac{d[j]}{dt} = k_{-2} \left(\sum_{i=2}^{j/2} [i][j-i] - [j] \sum_{i=2}^{N-j} [i] \right) + k_3 \left(\sum_{i=j+2}^{N} [i] - \left(\left\lfloor \frac{j}{2} \right\rfloor - 1 \right) [j] \right) - k_1 p([j] - [j-1]) + k_{-1}([j+1] - [j])$$

Number of equations: N

where *p* is the concentration of single platelets, [*i*] is the concentration of aggregates of size *i* , k_2 is the probability of new aggregate formation from two single platelets, k_1 in(is) the probability of another platelet attachment to an existing aggregate, k_{-1} is the probability of single platelet detachment from an aggregate, k_{-2} is the probability of formation of one aggregate from two existing ones, k_3 is the probability of an aggregate fragmenting into two, *N* is the maximum size to which an aggregate can grow. It consists of N differential equations, each of which represents the kinetics of aggregate of each size. The model was constructed in Python 3.6 and integrated using the LSODA. To investigate the role of size distribution with respect to parameter estimation we obtained parameters k_1, k_{-1}, k_2, k_{-2}, k_3 and p_0 for different N of the extended model. There were no differences between model parameters for N=10 and N=100 (Fig. S7), and so we can conclude that however size distribution throughout platelets may differ, but it does not affect the resulting aggregational curve.

Extended model parameters for experimental datasets given on Fig. S7. [plt] denotes concentration units.

Parameter	2.5 μM ADP	5 μM ADP	10 μM ADP	20 μM ADP
probability of a platelet attachment to an existing aggregate, k_1, 1/([plt]*s)	1.8<br><br>$8.3*10^{-3}$	$0.7*10^{-3}$<br><br>$3*10^{-8}$	$0.5*10^{-3}$<br><br>$3*10^{-8}$	$0.3*10^{-3}$<br><br>$1.02*10^{-14}$

probability of single platelet detachment from an aggregate, k_{-1}, 1/s	$0.4*10^{-3}$	$4*10^{-5}$	$4.2*10^{-5}$	$1.02*10^{-14}$
probability of one aggregate formation from two existing, k_{-2}, 1/([plt]*s)	$1.1*10^{-2}$	$9*10^{-2}$	$15*10^{-3}$	$12*10^{-3}$
probability of an aggregate fragmenting into two, k_3, 1/s	$2.2*10^{-6}$	$5*10^{-6}$	$5.9*10^{-6}$	$6.74*10^{-6}$
probability of a new aggregate formation, k_2, 1/([plt]*s)	1.97	1.6	1.49	1.20
impact of shape change, a, dimensionless	400	400	400	400
initial concentration of platelets, p_0, [plt]				

4. Supporting data on platelet aggregation potential

To assess comparative adhesiveness of single platelets during platelet aggregation, we performed aggregation test of Fura-Red loaded washed human platelets in presence of FITC-labeled fibrinogen (Fig. S9). The region of single platelets was chosen on the FSC-SSC dot plot as shown on Fig. S5A. The relative adhesiveness was assessed as the ratio of fluorescence of fibrinogen-FITC and Fura-Red for each event. As Fura-Red fluorescence in response to low ADP concentration changes only slightly and transiently (for the first 30 s), we considered the level of Fura-Red as the indicator of the number of cells in the flow event. As could be concluded from Fig. S9E, the fibrinogen binding to single platelets increases upon activation and does not decrease with time, while fibrinogen binding to aggregated platelets is significantly higher. However, decrease also has been observed (Fig. S10). Together these data indicate that the process of platelet disaggregation is determined by some instability in bonds between platelets in an aggregate. The details of this are shown in Fig. S10 that shows distributions of fibrinogen per platelet for platelets of different size. It could be concluded that the real single platelets have initial increase in fibrinogen binding upon activation, and then nothing changes. In contrast, aggregates have an increased (by an order of magnitude) fibrinogen binding that turns to the “normal activated” state upon disaggregation.

Supporting figures

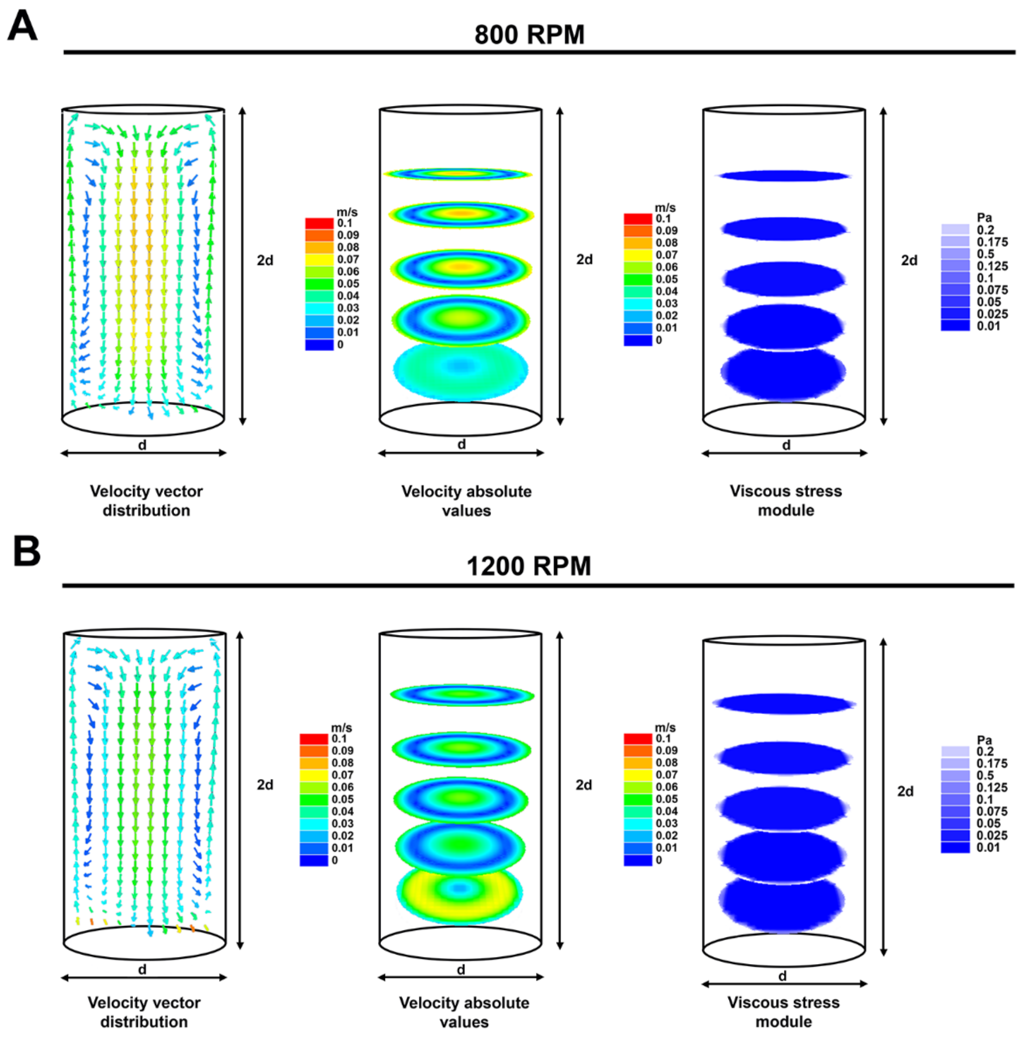

Figure S1. 3D-model of the flows in the aggregometric cuvette. Velocity vector distribution, absolute values and shear rates were calculated in FlowVision CFD Software. It can be observed that while in the middle part of the cuvette, the velocities as well as shear rates are similar, at the top and at the bottom of the cuvette, velocity vectors are curving, leading to the mixing of the suspension. The results for rpm 800 (A) and 1200 (B) do not significantly differ.

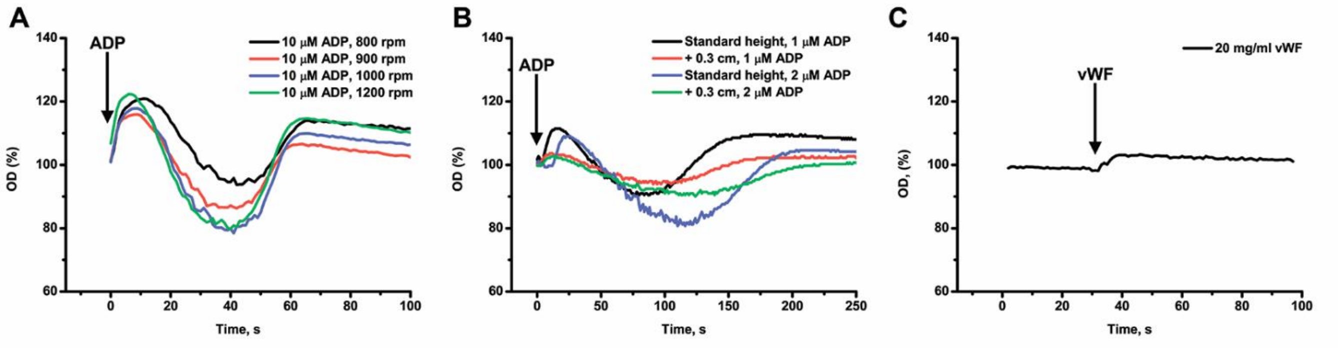

Figure S2. Aggregation of platelets in PRP. (A) The frequency of the stirring does not significantly affect platelet aggregation. (B) Platelet aggregation is significantly lower at the top of the cuvette than in the middle. (C) Platelet aggregation cannot be induced solely be the shear rate (1000 rpm), as has been demonstrated by addition of 20 mg/ml of von Willebrand Factor.

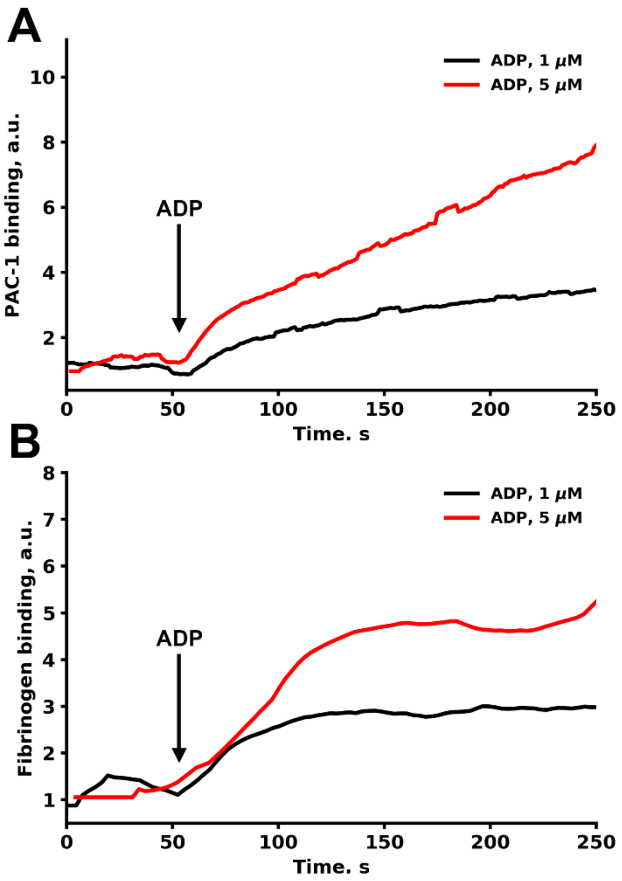

Figure S3. Platelet GPIIb-IIIa activation in response to ADP. Washed human platelets (1000/μl) in Tyrode's buffer (see Methods) in presence of calcium (2mM) were supplemented with FITC-labeled PAC-1 antibody (A) or fibrinogen (B) at time point "0" and analyzed by continues flow cytometry. After 60 s ADP at indicated final concentration was added into the solution.

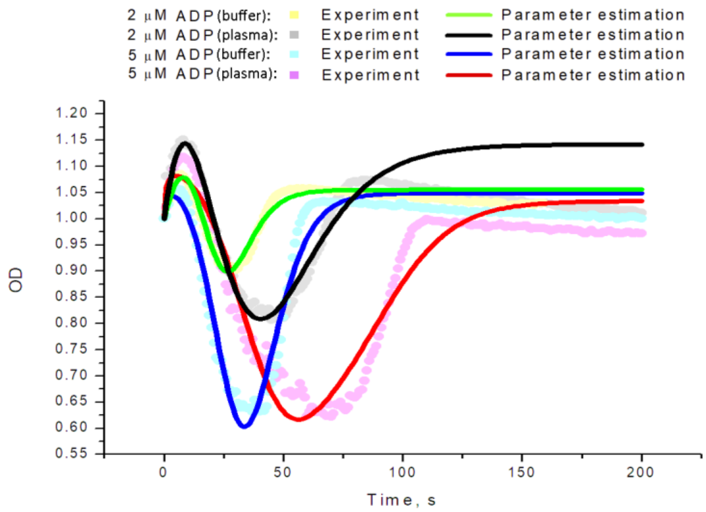

Figure S4. Comparison between platelet aggregation in plasma and in buffer by means of the mathematical model for platelet aggregometry. The estimation of five model parameters was conducted automatically by means of five different parameter estimation techniques implemented in COPASI software (see text). For each set of experimental data, parameters of the models were estimated independently. Experiments were performed with washed platelets in Tyrode's buffer ("buffer") or hirudinated platelet rich plasma ("plasma"), with platelet concentration adjusted to 200 000/µl, aggregation was induced with 2 or 5 µM of ADP, experimental curves were taken from the same blood sample of the same donor. The complete sets of parameter values for [washed platelets (2uM_ADP) – washed platelets (5uM_ADP) – prp (2uM_ADP) – prp(5uM_ADP))] were: k_1 = [1.43, 5, 1.1, 15]*10^{-6} 1/([plt]*s), k_{-1} = [3.7*10^{-15}, 4*10^{-10}, 4.3*10^{-8}, 0.92] 1/s, k_{-2} = [6.2, 5.7, 2.3, 4.7]*10^{-7} 1/([plt]*s), k_3 = [0.13, 0.12, 0.06, 0.07] 1/s, k_2 = [3.68, 0.38, 5.93, 0.08]*10^{-8} 1/([plt]*s), a = [1.05, 1.03, 1.15, 1.08].

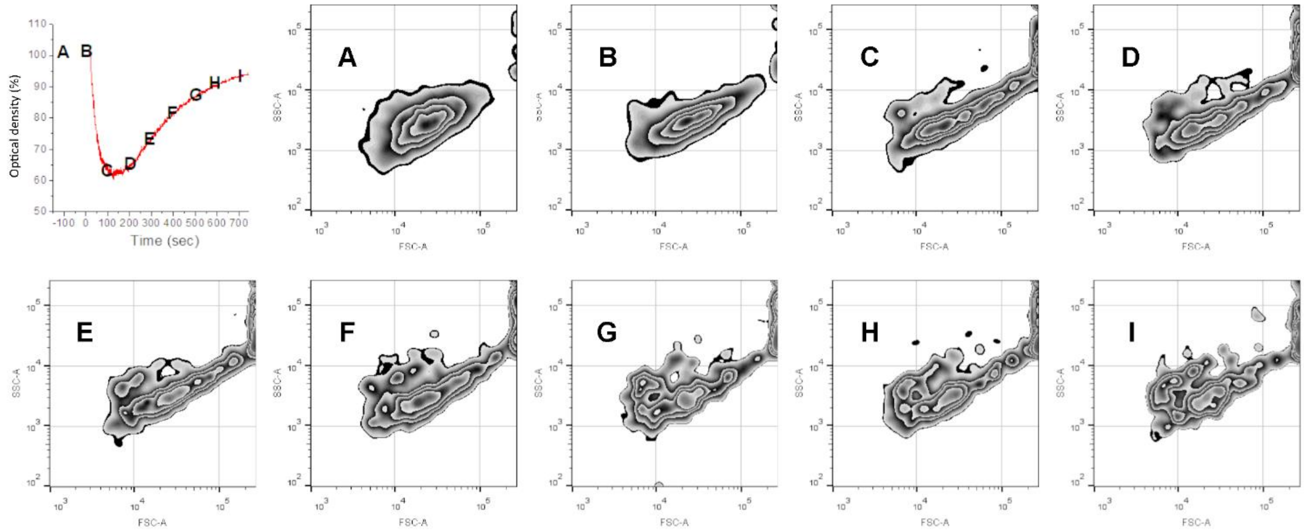

Figure S5. Flow cytometry estimation of side scattering/forward scattering of events during aggregometry test. Aggregation of platelet rich plasma stimulated with 2 µM ADP. Samples of the aggregating mixture were taken at points indicated as A, B, C … on the aggregation curve and immediately analyzed by flow cytometry.

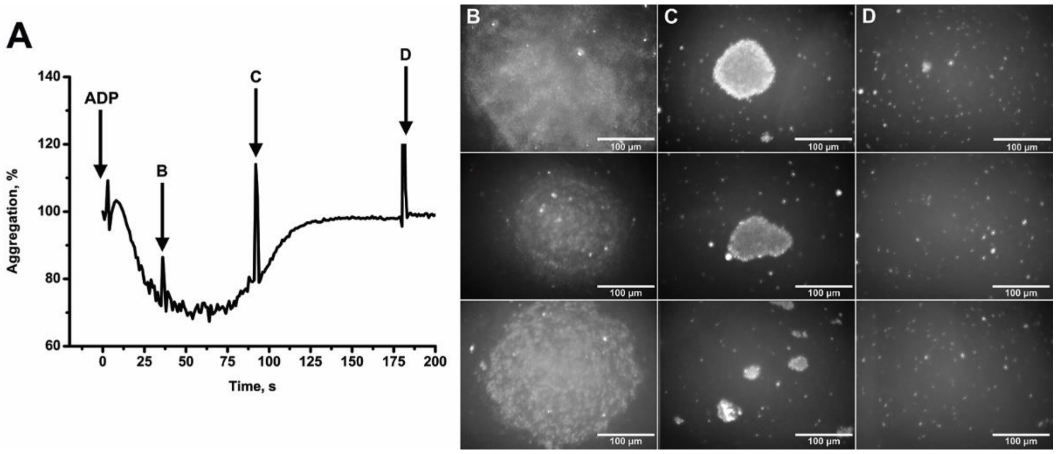

Figure S6. Observation of platelet aggregates by dark-field microscopy. Aggregation of platelet-rich plasma was simulated with 2uM of ADP. Samples of the aggregating mixture were taken at points indicated as B, C and D on the aggregation curve (A) and immediately analyzed by dark-field microscopy. Ten observations were conducted for each sample. For point (B) in average one large (> 500 um in diameter) and 2-5 medium (100 um) aggregates were observed. For point (C) one medium (100 um) and 2-8 small (20 um) aggregates were observed. For point (D) only small (<10 um) aggregates were observed. Typical aggregates out of n=48 are given.

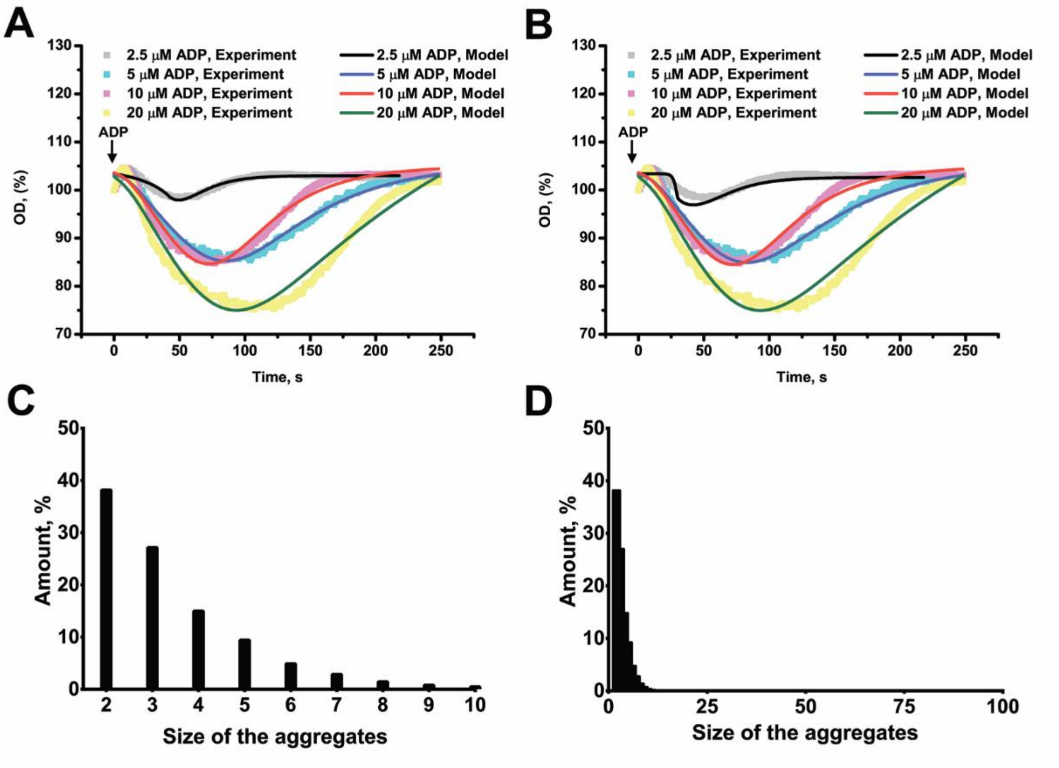

Figure S7. **Parameter estimation for extended model**. Estimation of six model parameters was conducted automatically by means of five different parameter estimation techniques implemented in COPASI software (see text) for maximum aggregate size N=10 (A) and manually using Python 3.6 for restriction N=100 (B). (A), (B) Washed platelets, stimulation with 2.5, 5, 10 or 20 μM of ADP, experimental curves were taken from the same blood sample of the same donor. For fitted curve for simulation with 20uM of ADP at minimum point (t=100) size distribution was calculated. Histogram represents percent of aggregates of each size for N=10 (C) and N=100 (D).

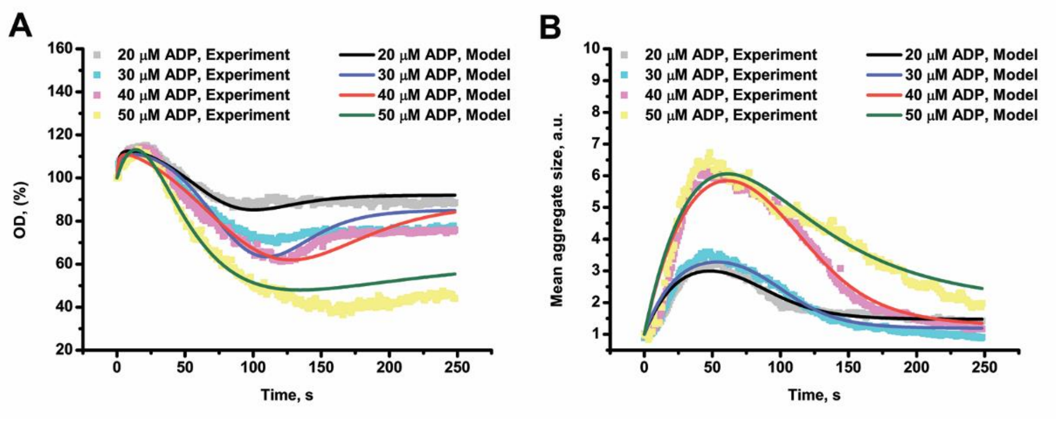

Figure S8. Platelet responses to high concentrations of ADP. Activation by ADP of platelet-rich-plasma with low platelet concentration (100 000/ul). Parameter estimation for platelet aggregometry data obtained by Biola. Estimation of five model parameters and initial platelet concentration was conducted automatically either with both light transmission curve and mean aggregate size as input experimental data. The complete sets of parameter values for [20uM, 30 uM, 40 uM, 50uM] ADP were: k_1 = [2.8, 1.2, 2.1, 2.6]*10^{-3} 1/([plt]*s), k_{-1} = [0.16, 1.7*10^{-17}, 0.06, 3.2*10^{-12}] 1/s, k_{-2} = [11, 3.7, 4.1, 5.9]*10^{-4} 1/([plt]*s), k_3 = [0.040, 0.042, 0.027, 0.022] 1/s, k_2 = [1.7*10^{-17}, 2.1*10^{-16}, 2.3*10^{-16}, 4.6*10^{-6}] 1/([plt]*s), a = 1.15, p_0 = 100.

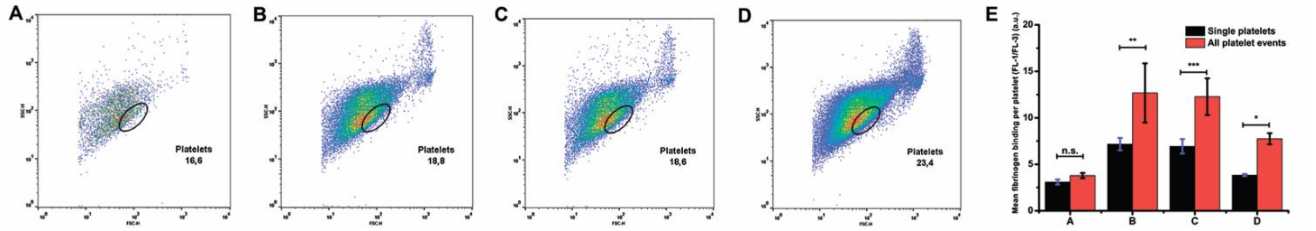

Figure S9. Flow cytometry estimation of platelet adhesiveness during aggregometry test. Aggregation of washed loaded with Fura Red human platelets in Tyrode's calcium buffer ($1.8 \cdot 10^5$/μl) in the presence of 4 mg/ml FITC-labeled human fibrinogen was stimulated with 5 μM ADP. Samples of the aggregating mixture were taken at -30 s (A), 2 min (B), 5 min (C) and 10 min (D) after addition of ADP and then analyzed by flow cytometry. (E) Relative fibrinogen binding to single platelets calculated as ratio of the amount of fibrinogen (FL-1, fibrinogen-FITC) to the size of the aggregate (FL-3, Fura-Red). One typical aggregation curve out of n = 3 for this donor, similar results were obtained for n = 2 another donors. Statistical analysis performed with Mann-Whitney test, (*) indicates $p<0.05$, (**) indicates $p<0.01$, (***) indicates $p<0.001$.

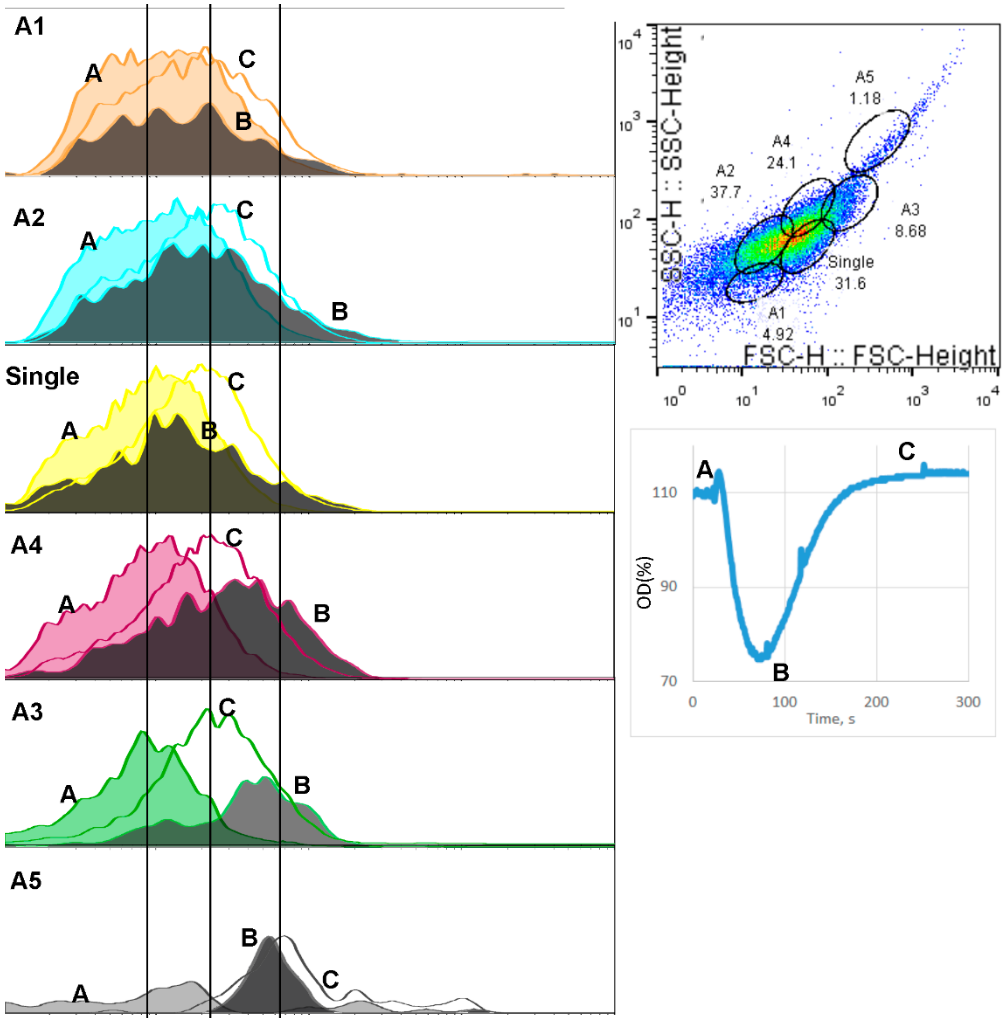

Figure S10. Flow cytometry estimation of platelet aggregation potential during aggregometry test in different aggregate size regions. Aggregation of washed loaded with Fura Red human platelets in Tyrode's calcium buffer ($3 \cdot 10^5/\mu l$) in the presence of 200 µg/ml FITC-labeled human fibrinogen was stimulated with 2.5 µM ADP. Samples of the aggregating mixture were taken at indicated points (A, B, C) and immediately analyzed by flow cytometry. Relative fibrinogen binding to platelets were measured as ratio of signals in FL-1 (fibrinogen-FITC) and FL-3 (Fura-Red). Histograms of relative fibrinogen binding to platelets from indicated regions (Ai) are given.

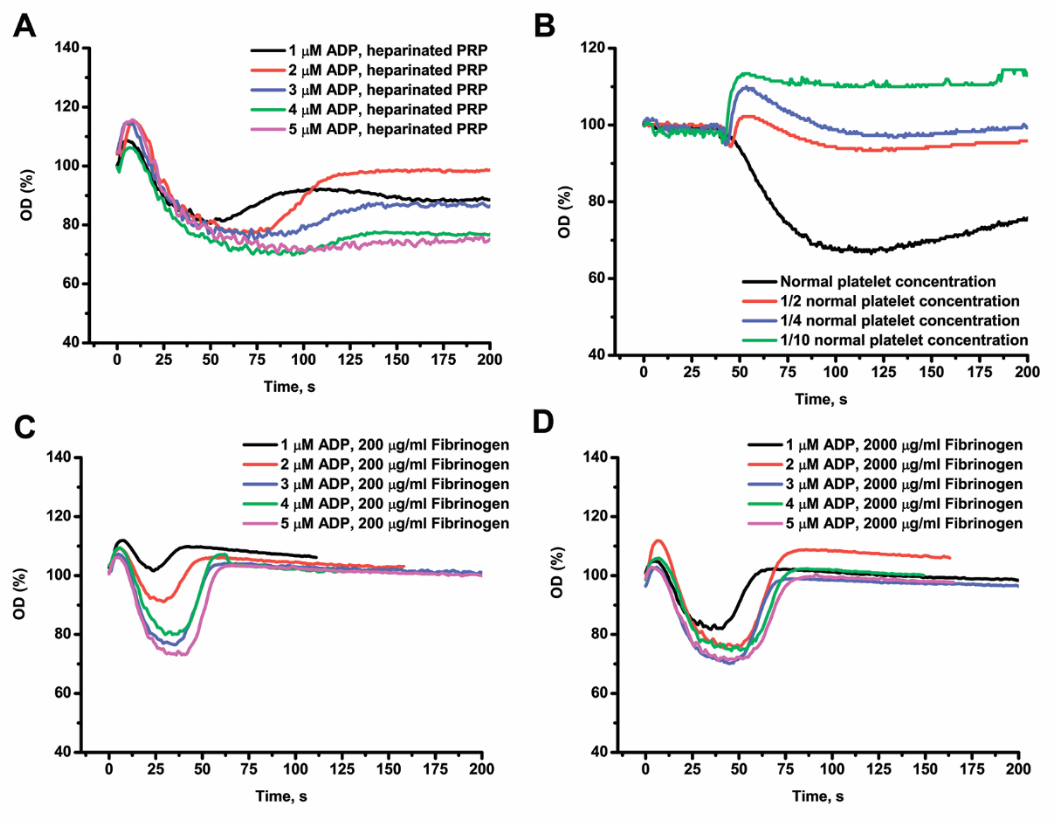

Figure S11. Platelet responses to ADP for PRP and washed platelets. (A) Blood was collected into tubes with Li-heparin. Then PRP was obtained and platelet count was adjusted as described in Materials and Methods. Activation by different concentrations of ADP (1-5 μM). Concentration of platelets in both PRP and WP was adjusted to 200 000/μl; (B) washed platelet responses to ADP (5 μM) for different platelet concentrations in the presence of fibrinogen (300ug/ml); (C,D) washed platelet suspension in the presence of different concentrations of fibrinogen (200ug/ml (C) and 2000ug/ml (D)).
